# Incorporation of Ornamental Stone Waste in the Manufacturing of Red Ceramics

**DOI:** 10.3390/ma15165635

**Published:** 2022-08-16

**Authors:** Mônica Castoldi Borlini Gadioli, Mariane Costalonga de Aguiar, Francisco Wilson Hollanda Vidal, Maria Angelica Kramer Sant’Ana, Kayrone Marvila de Almeida, Ana Júlia Nali Giori

**Affiliations:** Centre for Mineral Technology, State of Espirito Santo Branch-CETEM/NRES, Cachoeiro de Itapemirim 29311-970, ES, Brazil

**Keywords:** red ceramic, ornamental stone, waste

## Abstract

Brazil is one of the largest producers of ornamental stones in the world. The state of Espírito Santo has considerable social and economic relevance in the production of ornamental stones, particularly in exportation and the jobs that are directly related to this industry. The objectives of this work were to evaluate the effect of the incorporation of ornamental stone waste on the physical and mechanical properties of red ceramic manufactured using clays and waste from the state of Espírito Santo, and to collaborate to regulate the use of this ornamental stone waste in the ceramic industry when manufacturing products. Ornamental stone wastes were incorporated into the ceramic mass in the following proportions: 0, 10, 20, 30, 40 and 50% by weight. In the elaborated compositions, specimens were prepared by extrusion and were fired at 1050 °C and 1100 °C. After firing, the physical and mechanical properties of the material were analyzed using density, water absorption, porosity, linear shrinkage and mechanical strength. The results indicated an improvement in the properties of the ceramics with the addition of the waste by mass for the two temperatures. Therefore, the lower temperature (1050 °C) can be used to sinter the materials produced whilst obtaining satisfactory results and saving electrical energy. Ornamental stone waste has very promising applications in the ceramic industry.

## 1. Introduction

Brazil is a large producer and exporter of ornamental stones. In the first quarter of 2021, Brazilian exports of natural stone materials for ornamentation and cladding totaled US$572 million (R$3.1 billion) and 1.12 million t, with a positive variation of 43.83% in US dollars and 20.42% in physical volume compared to the same period in 2020 [1]. Espírito Santo is the main Brazilian exporting state of ornamental stones, with sales of US$827.7 million (i.e., 82% of the country’s sales) and 1.66 million t (i.e., 77% of the total physical volume) [2].

As a result of the large production of ornamental stones, the sector generates large amounts of solid waste, resulting from both extraction and processing. Mining is one of the sectors that generates the most waste as it has low utilization rates during production. In Brazil, it is estimated that the utilization rate from extraction to the production of slabs is approximately 17%. Therefore, for every 100 t of extracted stones, on average only 17 t will become slabs for sale [3]. 

To boost sustainable industrial development, it is necessary to achieve good management of solid waste. With this in mind, in 1972 the United Nations Conference on Development and the Human Environment took place in Stockholm. One of the focuses of the conference was pollution due to industrialization and the excessive use of natural resources [4]. The main objective of the Stockholm Declaration was to address and discuss environmental problems in the world. Since the conference, concern about waste management has been increasing. This is due to the huge amount of waste that is generated annually by industries.

In recent years, researchers have sought to apply industrial waste in the manufacturing of new products. By reusing this waste, it is possible to significantly contribute to the precepts of sustainability and the circular economy. Recent studies show that discarded waste (e.g., rice straw) can be utilized in agriculture, energy generation, the creation of adsorbents and the creation of new products [5]. With high population demand and the growing industrial sector, it is important to create sustainable technologies that promote the substitution of non-renewable natural resources. Activated charcoal is a high-cost non-renewable energy source. The replacement of activated charcoal with biomass, such as banana peel, wheat straw and other materials, can contribute to environmental conservation and reductions in waste disposal [6]. Other research shows that alternative materials are being used in order to reduce waste in industries. One of the alternatives may be the use of biomass as a renewable energy source, thereby contributing to the sustainable bioeconomy [7].

During the production of ornamental stones, large amounts of waste are generated. During processing, approximately 26% of a block of stone is transformed into fine waste [8]. Brazil produces about 2.5 million tons of this waste annually [9]. Since the 1990s, ornamental stone waste has been studied for possible applications, such as in ceramic artifacts [10], or for incorporation in the manufacturing of soil-cement bricks [11]. Currently, there are several studies that also seek to apply these wastes. Finding an alternative for their application contributes to the sustainable development of the stone sector. The application of these wastes in new products strengthens the three principles of the sustainability tripod, which are social, environmental and economic factors.

In this context, the dimension stone sector faces the challenge of disposing a substantial amount of waste throughout the process and doing so properly, as the improper disposal of waste can cause environmental impacts, including soil and water pollution and damage to aquatic life [12].

Waste discarded from the ornamental stone industry has mineralogical and chemical characteristics that can increase the quality and favor the manufacturing of various materials. Thus, researchers in different areas of knowledge have been looking for alternatives to insert waste into the production cycle of new materials so that the sector’s economy becomes increasingly sustainable and shifts from linear to circular [3,13].

In this sense, the addition of wastes in the formulation of new materials helps to mitigate the environmental impacts of production, maximize the use of non-renewable natural resources and subsequently aids the economic development of the sectors involved.

Research has been carried out for many materials, including: polymers, in which waste acts as a filler and research had the objective of improving their mechanical and thermal properties [14]; asphalt paving to increase stability, hardness and tensile strength [15]; glass to aid in vitreous phase formation, where glass was used as a lattice former and modifier, as a colorant and as a reducer of viscosity and melting point [16,17]; and concrete and aggregates, which were used as fillers and research had the objective of increasing the resistance to compression, abrasion and to chlorides and sulfates [3,18].

Another important sector for the use of ornamental stone waste is ceramics, where the waste can help to reduce the temperature of vitrification and burning, allow greater control of linear contraction and reduce porosity.

Red ceramics are an important sector for the national economy. There are approximately 6903 companies in Brazil, most of which are small- and medium-sized, and generate around R$18 billion annually, 293,000 direct jobs and 900,000 indirect jobs. The red ceramic industry represents 4.8% of the civil construction industries, and about 90% of masonry and roofing are built with these materials. Among the Brazilian regions, the Southeast is the region with the highest representation, with 44.38% of national production [19,20,21].

Nowadays, ornamental stone processing waste is deposited directly in landfills, which greatly impacts the environment. Since 1990, alternative applications of this waste have been studied. Several research studies have investigated the incorporation of this waste in the production of red ceramic artifacts. However, there is still no current regulation in Brazil that certifies the use of this waste. Within this context, the Centre for Mineral Technology (CETEM), together with the Fundação de Amparo à Pesquisa e Inovação do Espírito Santo-FAPES, developed a standardized project for the use of waste obtained from the processing of ornamental stone in red ceramic artifacts. The project contributes to reducing the environmental impact generated by the disposal of waste, reducing the consumption of raw materials in the manufacturing of artifacts, environmental education, cost reduction in the production of red ceramic artifacts, the possibility of adding value to the waste of ornamental stones and, consequently, sustainable development of the Brazilian ornamental stone and civil construction sector.

Based on the above, the objective of this work was to evaluate the effect of incorporating ornamental stone waste on the physical and mechanical properties of red ceramics made with clays and waste from the state of Espírito Santo, Brazil and, subsequently, to collaborate in order to standardize the use of this waste in the manufacturing of ceramics, thereby contributing to the manufacturing of products with ornamental stone wastes. This work is of great importance for Brazil, as it may contribute to the regulation of the use of waste by the red ceramic industry through normative instructions or norms.

## 2. Materials and Methods

### 2.1. Material Used

The raw materials used in this work were ceramic mass from São Roque do Canaã-ES and granite waste from the sawing process obtained using a multiwire gangsaw in the municipality of Cachoeiro de Itapemirim-ES, Brazil.

**Figure 1:**
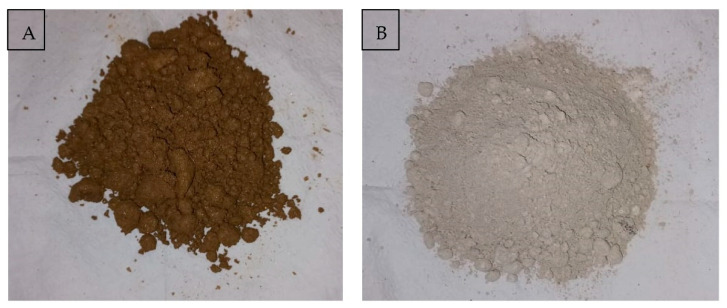
Raw materials used to manufacture the ceramic artifact: (**A**) ceramic mass, (**B**) ornamental stone waste.

Figure 1 shows the raw materials that were used to manufacture the ceramic artifact.

### 2.2. Characterization of Raw Materials

X-ray diffraction was determined by the powder method. Data were collected in a Bruker D4 Endeavor under the following operating conditions: Co Kα radiation (35 kV/40 mA); a goniometer speed of 0.02° 2θ per step with a counting time of 1 s per step; and collection from 5 to 80° 2θ. Qualitative spectrum interpretations were performed by comparison with standards contained in the PDF02 database (ICDD, 2006) in Bruker AXS software “Diffrac Plus”.

The results presented are expressed in % and are the means of three readings determined by semi-quantitative analysis (standardless) in an X-ray fluorescence spectrometer—(WDS-1), model AxiosMax (Panalytical).

For the determination of loss on ignition, the LOI of the samples was made in Mufla. Aliquots of each sample were separated, placed in the muffle at 1000 °C for 16 h and, after cooling, were weighed to verify the loss due to ignition.

The FRX equipment analyzes samples on a global calibration curve, with a detection limit for values greater than 0.1%. Therefore, values less than 0.1% were not reported. ND—element not detected by FRX in the analyzed sample.

The distribution of the raw material (stone waste) was obtained from the Malvern Mastersizer equipment using the low angle laser light scattering technique, known generically as “light scattering”.

Plasticity was obtained according to the standards of ABNT NBR 7180 (1984c) [22] and ABNT NBR 6459 (1984d) [23]. The Atterberg plasticity index (IP) is given by:

IP = LL − LP

where the plasticity limit (LP) is the water content (expressed in %) of the dry pulp weight at 110 °C, above which the clayey mass can be molded into cylinders about 3 to 4 mm in diameter and 15 cm long. The liquidity limit (LL) is the water content (expressed in %) of the dry pulp weight at 110 °C, above which the pulp flows as a liquid when slightly agitated. These tests were carried out in the civil engineering laboratory of the State University of Northern Fluminense Darcy Ribeiro-UENF.

### 2.3. Preparation of Formulations for Extrusion

This step consisted of the formulation of ceramic mass compositions with the incorporation of stone waste from the multiwire gangsaw. The red ceramic mass compositions were prepared using granite waste in the amounts of 0, 20, 30, 40 and 50%. The homogenization of raw materials was carried out in a ball mill. Table 1 presents the studied compositions.

### 2.4. Processing of Specimens Made by Extrusion

The specimens were formed by vacuum extrusion, in dimensions 120 × 30 × 18 mm, in a laboratory extruder of the brand Verdés from the Civil Engineering Laboratory (LECIV/UENF). The specimens were air-dried and were then dried in an oven at 110 °C until they reached a constant weight.

The dimensions of the specimens were measured at two timepoints: after removal from the extruder and after removal from the oven, with the aid of a MITUTUYO digital caliper (resolution ± 0.01 mm), and were weighed using a SHIMADZU digital scale (model UX6200H, accuracy 0.01 g).

Firing at temperatures of 1050 °C and 1100 °C was carried out in a laboratory furnace of the Maitec FL 1300 muffle type, used at a heating rate of 2 °C/min. The specimens were maintained at these temperatures for 180 min and were cooled by natural convection once the oven was turned off.

**Figure 2:**
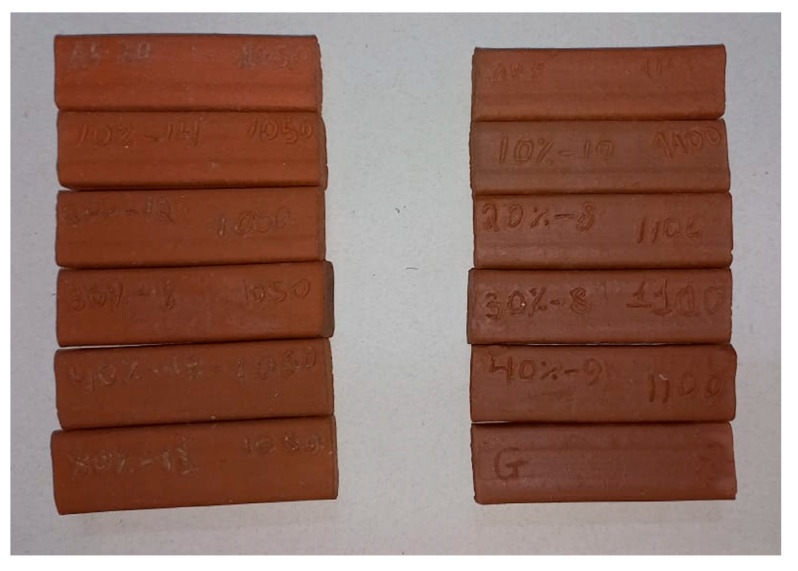
Ceramic specimens made from clay and ornamental stone waste.

Figure 2 shows the specimens after firing.

### 2.5. Physical and Mechanical Tests of Specimens

To determine the apparent density of green, dry and burned pieces, the dimensional method was used in accordance with ASTM C 373-72 (1977) [24].

Determination of the apparent porosity (PA) of the ceramic bodies was made in accordance with ASTM C373-88 (1994) [25].

The linear shrinkage of the burned pieces (RL) was determined with the aid of a MITUTOYO digital caliper (resolution ± 0.01 mm).

The water absorption test was performed in accordance with ABNT 15270-2 (2017) [26]. The specimens were weighed, placed in a container with water at room temperature and were kept for 24 h. Then, the surface water from each piece was removed and the mass of each piece was recorded.

The flexural strength (σ) was evaluated using the three-point bending test, in accordance with ASTM C674-77 (1977b) [27].

The environmental analysis of the ornamental stone waste involved leaching and solubilization tests at Tommasi Ambiental, Serra (ES). The reference standards were: NBR 10004—classification of solid waste (ABNT, 2004a); NBR 10005—leaching test (ABNT, 2004b); and NBR 10006—solubilization test (ABNT, 2004c) [28,29,30].

## 3. Results and Discussion

### 3.1. Chemical Characterization

Table 2 shows the chemical composition of the raw materials. The ceramic mass was predominantly made up of SiO_2_ and Al_2_O_3_, whereas the granite waste had a very high content of SiO_2_. The Si and Al oxides are mostly associated, forming the structures of aluminosilicates such as kaolinite. The content of alkaline flux oxides in clays was also comparatively low. These oxides contribute to the formation of the liquid phase during firing, enabling a reduction in porosity. Note also that the ceramic mass had a high content of iron oxide. Iron compounds are mainly responsible for the reddish color of ceramics after firing. The high loss to fire, determined by loss on ignition (LOI) of the clayey ceramic mass, indicates the significant presence of kaolinite, which theoretically has a loss to fire of 14% [31].

The chemical composition of clay provides important information that aids the formulation of ceramic masses. Kaolinitic clay has a very small amount of fluxing oxides, resulting in the slow formation of the liquid phase of ceramic. Thus, the addition of fluxing oxides is required in order to improve the technological properties of the ceramic.

On the other hand, dimension stone wastes have a high content of alkaline oxides (Na_2_O and K_2_O) and alkaline earth oxides (CaO and MgO), which are fluxing oxides and, therefore, help the formation of the liquid phase in ceramic firing [32,33,34,35,36,37,38]. CaO and MgO are widely used in the formulation of ceramic masses for porous coatings. They react with amorphous phases and form crystalline phases that are more stable in the presence of moisture.

In the chemical composition of the granite waste, significant amounts of alkaline oxides (K_2_O and Na_2_O), equal to 9.7% in weight, were observed, which act as fluxes. Despite the process of beneficiation using the multiwire gangsaw technology, the waste presented a large amount of Fe_2_O_3_. This value is associated with the high iron content of the stone itself when it had the same Fe_2_O_3_ content as the waste (5.8%).

### 3.2. Mineral Characterization

**Figure 3:**
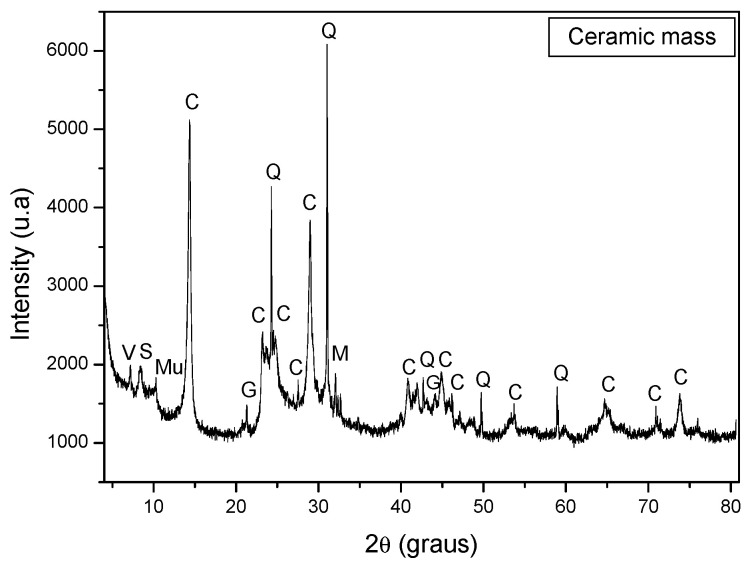
X-ray diffractogram of the ceramic mass. C = Kaolinite, Q = Quartz, G = Gibbysite, M = Microcline, Mu = Muscovite, S = Sepiolite, V = Vermiculite.

Figure 3 shows the diffractogram of the ceramic mass. Note the presence of kaolinite (2SiO_2_.Al_2_O_3_.2H_2_O), quartz (SiO_2_), gibbsite (Al(OH)_3_), microcline (KAlSi_3_O_8_), muscovite (K_2_O.3Al_2_O_3_.6SiO_2_.2H_2_O), sepiolite (Mg₄Si₆O₁₅ (OH)_6_·6H₂O) and vermiculite (MgFe, Al)₃(Al, Si)₄O₁₀(OH)₂.4H₂O).

**Figure 4:**
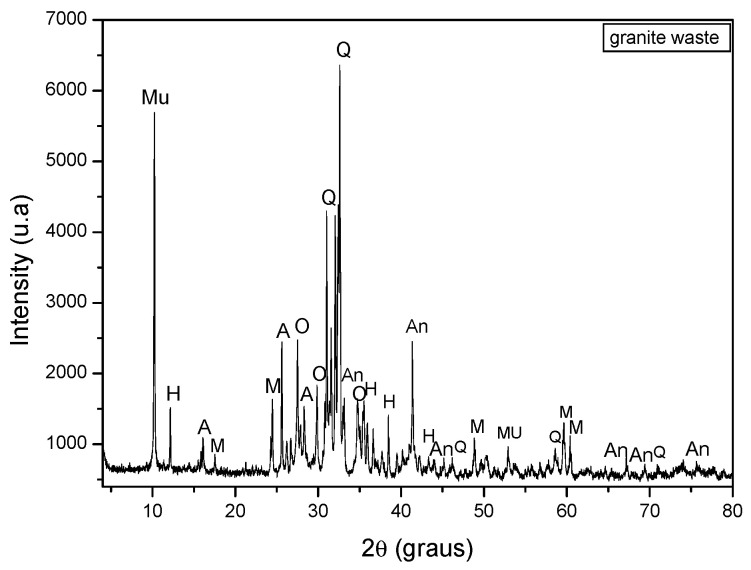
X-ray diffractogram of the multiwire gangsaw waste. A = Albite, An = Anortite, H = Hornblende, M = Microcline, Mu = Muscovite, O = Orthoclase, Q = Quartz.

Figure 4 shows the diffractogram of the multiwire gangsaw waste corresponding to quartz (SiO_2_), albite (NaAlSi_3_O_8_), anorthite (CaAl_2_Si_2_O_8_), hornblende (Ca,Na)2-3(Mg,Fe,Al)_5_(Al,Si)_8_O_22_(OH,F)_2_), microcline (KAlSi_3_O_8_), muscovite (KAl_2_(Si_3_Al)O_10_(OH,F))_2_ and orthoclase (KAlSi_3_O_8_).

The presence of the clay mineral kaolinite was observed in the ceramic mass. This is the mineral responsible for the development of plasticity. Quartz is the main impurity present in clays, acting as a non-plastic and inert raw material during firing, increasing the permeability of the green piece and controlling drying and firing shrinkage. Gibbsite contributes to the increased refractoriness of clays and mass loss during firing. Muscovite mica is a mineral with lamellar morphology that can cause the appearance of defects in ceramic pieces. As long as it has a reduced particle size, muscovite mica can act as a flux due to the presence of alkaline oxides.

### 3.3. Waste Granulometry

**Figure 5:**
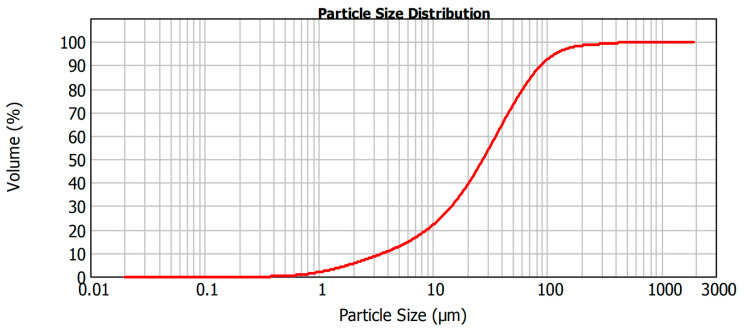
Multiwire gangsaw waste particle size distribution (% by weight).

Figure 5 shows the granulometry of the multiwire gangsaw waste. In the multiwire gangsaw waste, 10% of the particles were below 3.681 µm (thereby confirming that the material was not plastic), 50% were below 27.368 µm and 90% were below 87.931 µm.

The dimension stone waste had a wide granulometric range, which contributed to better packing of the particles and, consequently, better properties of the final product.

### 3.4. Plasticity

Table 3 shows the plasticity of the studied compositions, determined using the Atterberg limits. Obtaining plasticity in clay is of fundamental importance for its use and, based on this property, many ceramic products have been manufactured since ancient times.

**Figure 6:**
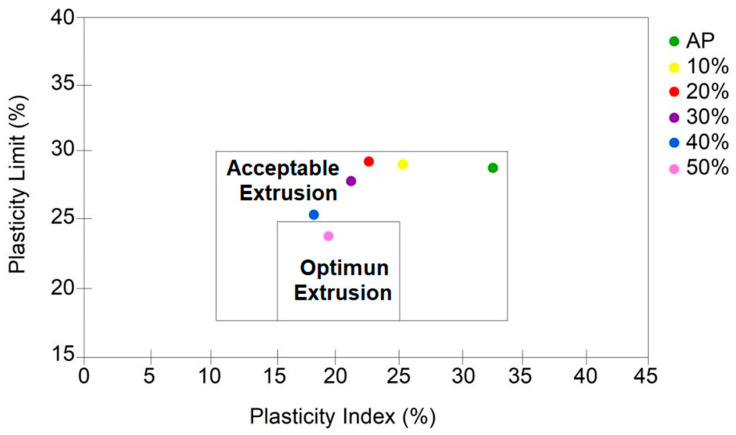
Extrusion prognosis using the Atterberg limits of ceramic masses.

Figure 6 shows a graph drawn from the Atterberg Limits. The formulation with pure clay (PC) was positioned within the limit at the limit of the acceptable region. This indicated that the ceramic mass presented good workability/plasticity. Masses with the addition of 10% GW, 20% GW, 30% GW and 40% GW also fell into the acceptable extrusion limit region. Formulations with the addition of 50% GW was positioned at the optimal extrusion limit. This means that the waste improved the workability of the ceramic mass.

### 3.5. Density

**Figure 7:**
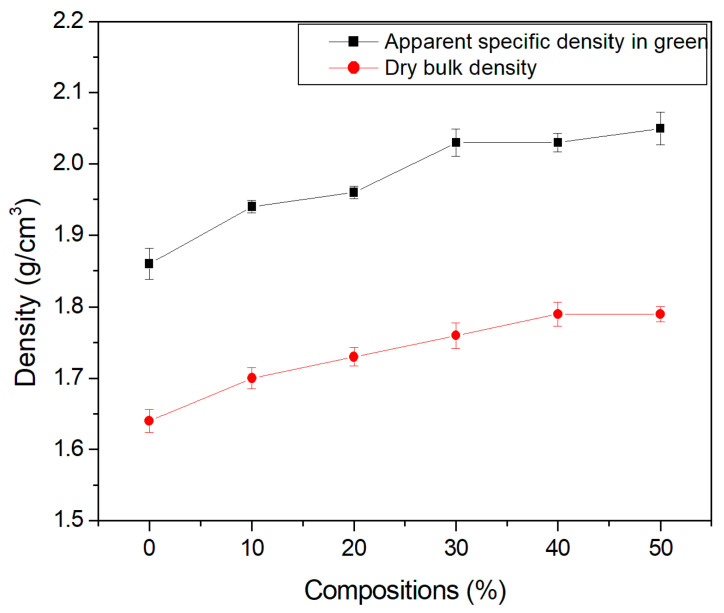
Green and dry specific density of the compositions.

**Figure 8:**
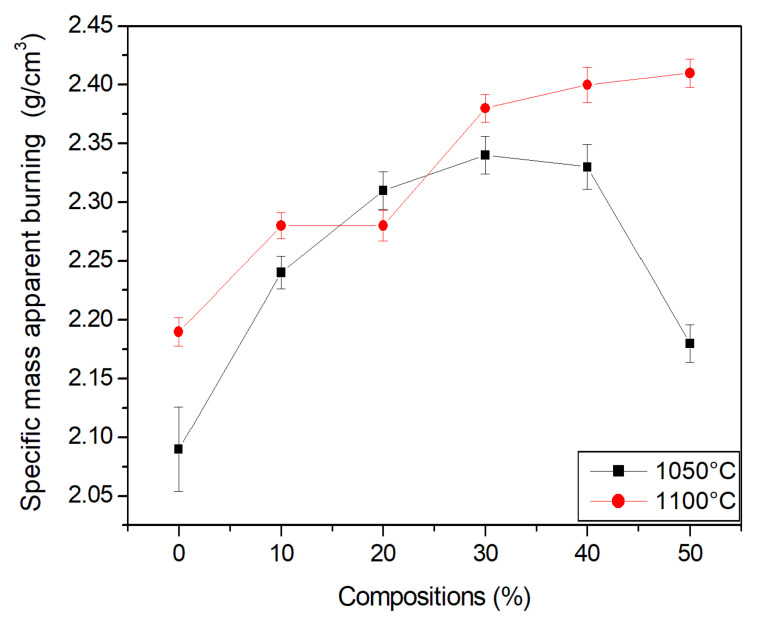
Specific mass apparent burning of the compositions.

Figure 7 and Figure 8 show the densities of the studied ceramics. Density was determined using the geometric method, with the aid of a caliper and scale. The dry density of the compositions present values lower than those observed for the density in green. This is attributed to the loss of mass of the material, which occurred as water was lost during the processing of the specimen.

The apparent density of the specimens increased with the temperature, which reaffirms a previous study [39] in which temperature was found to be an important parameter in sintering. Note that there was an increase in density with the incorporation of the granite waste, thereby improving the packing of the particles. This is beneficial in terms of reducing shrinkage and favoring particle consolidation during firing.

A slight increase in the density and specific mass of the ceramic material at temperatures of 1050 °C and 1100 °C was observed, indicating that there was better packing of the particles in the specimens. Improved packing can be beneficial as it may contribute to better consolidation of the particles in the burning stage, thereby improving technological properties.

### 3.6. Linear Shrinkage

**Figure 9:**
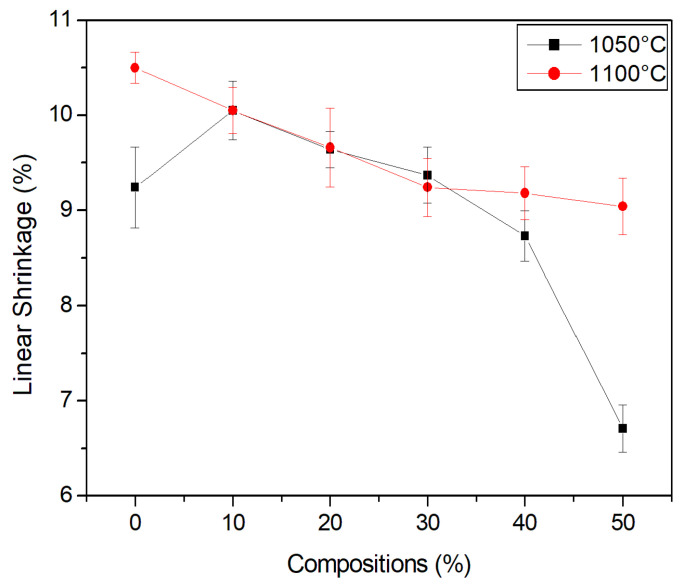
Linear shrinkage of the compositions.

Figure 9 shows the linear shrinkage of the ceramics. Sintering tends to decrease the surface area of the body, which includes a decrease in pore volume. Consequently, the structure contracts.

Note that there was an increase in linear shrinkage in the compositions, in relation to the evaluated burning temperatures. This occurs due to evolution of the material’s densification and, consequently, a decrease in porosity. Furthermore, an increase in shrinkage makes the risk of heating cracks greater.

### 3.7. Absorption

**Figure 10:**
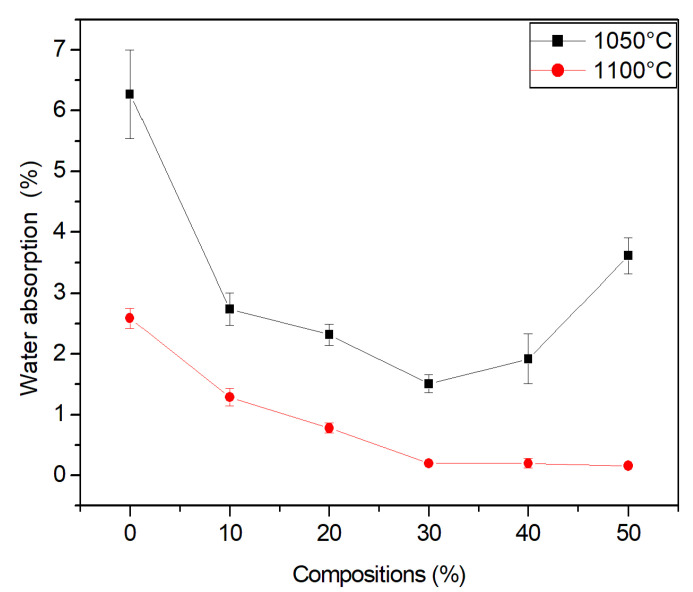
Water absorption of the ceramic material.

Figure 10 shows the water absorption of the studied compositions. Note that granite waste had a tendency to reduce water absorption at all of the temperatures studied. This was due to improved packing and reduced mass loss during firing. The reduction in water absorption with a decrease in porosity occurred as a consequence of the sintering reactions.

This is associated with the melting action of the waste, known as liquid phase sintering [40], filling the pores and densifying the ceramic body. As the temperature increases, there is greater formation of a liquid phase. However, as seen above, this involves further retraction.

For sealing blocks, according to standard NBR 15270-1 (2017), the water absorption index must not be lower than 8% or greater than 25% [41]. The standard NBR 15310 (2009) indicates that the maximum admissible limit of water absorption for ceramic tiles is 20% [42].

### 3.8. Porosity

**Figure 11:**
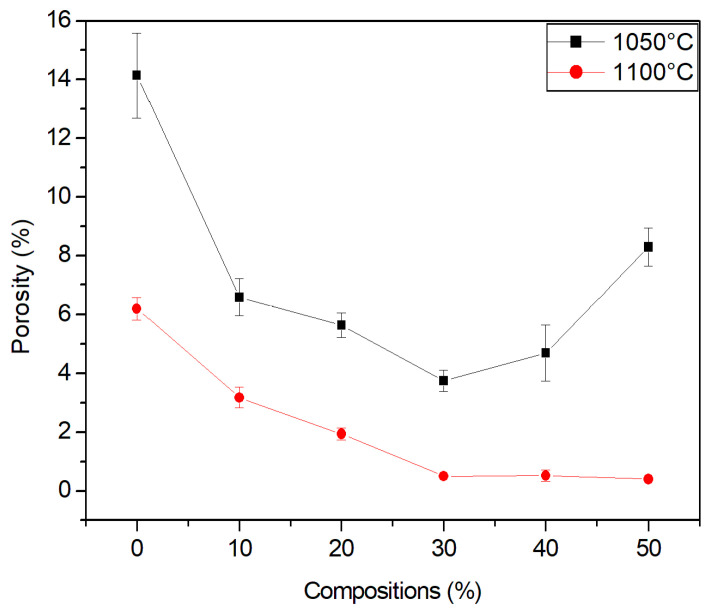
Porosity of ceramic material.

Figure 11 shows the porosity of the studied compositions. The apparent porosity of red ceramic pieces was influenced both by the firing temperature and by the percentage of granite waste incorporated into the ceramic material.

The ceramic material behaved in a similar way in both the water absorption and porosity tests, which corroborates the open porosity of the ceramic pieces.

### 3.9. Flexural Strength

**Figure 12:**
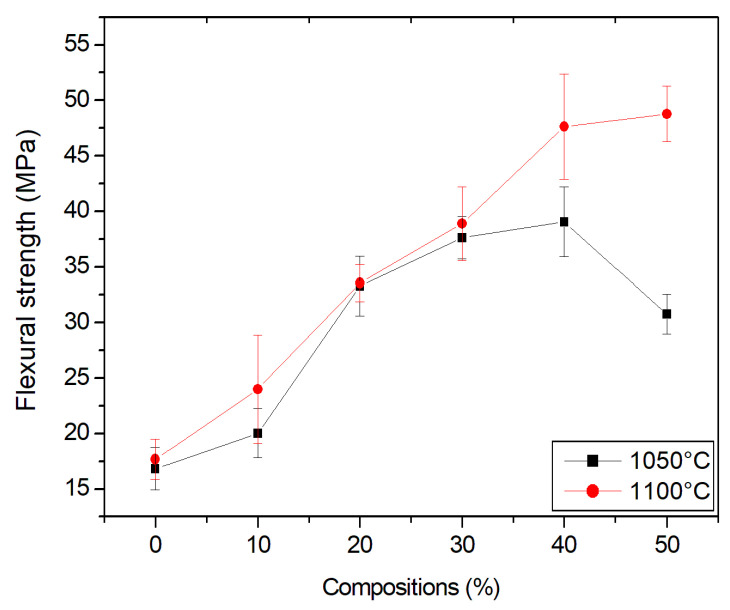
Ceramic material flexural strength.

Figure 12 shows the flexural strength of the studied compositions. Note that the mechanical resistance increased with increasing temperature for the specimens made with clay and granite waste. This was due to the sintering mechanisms that enabled greater formation of the liquid phase, thus reducing the porosity of the material and promoting better particle consolidation. The evolution of flexural strength with increasing temperature demonstrates that sintering occurred.

Due to the reduction in water absorption, an increase in the flexural strength of the clay with the addition of ornamental stone waste was expected. Water absorption is associated with the open porosity of the ceramic material and, therefore, with the interior microstructural characteristics of the pieces.

The ceramics that were made with ornamental stone waste showed better technological properties than the ceramics made without waste. Note that, at 1050 °C, the strength of the ceramic composed of 50% waste decreased. However, it remained higher than the ceramic made without waste. This was different for the ceramic composed of 50% waste that was fired at 1100 °C. Another factor that must have significantly influenced this decrease was quartz, a mineral that makes up the waste, which probably acted as an inert material and may have contributed to the generation of micro cracks [43].

According to standard NBR 15310 (2009), the minimum strength limit is 1300 newtons for Roman tiles and 1000 newtons for other tiles. All specimens had a force in newtons above that which is deemed acceptable by the standard [44].

The present work tested waste incorporation percentages of up to 50% in parts by extrusion and promising results were obtained. Previous studies have already indicated that dimension stone waste could have a favorable behavior in compositions of up to 40% for extrusion and 50% for pressing [34,45]. This study demonstrates the possibility of waste incorporation of up to 50% with extrusion as well.

### 3.10. Leaching Test

Table 4 presents the results of the ornamental stones waste (multiwire gangsaw) leaching test. Comparing the results obtained with the maximum values allowed by NBR 10004, the parameters satisfy the allowed limits. Thus, the waste does not present toxicity. Therefore, this material is classified as class II (i.e., non-hazardous waste).

### 3.11. Solubilization Test

Table 5 presents the results of the ornamental stone waste solubilization test (multiwire gangsaw). Comparing the results obtained with the maximum values allowed by NBR 10004, all parameters do not exceed the maximum allowed limits. Therefore, the residue can be classified as class II B (i.e., inert).

## 4. Conclusions

In this work involving the manufacturing of ceramic material incorporated with ornamental stone waste, the following conclusions were reached:

- Granite waste has characteristics suitable for use by the red ceramic segment, such as fine particle size.
- The waste improves the workability/plasticity of the clay, enabling adjustment in the ceramic shaping step.
- The waste has significant percentages of alkaline and alkaline earth oxides that act as fluxes during the firing stage.
- There was an increase in the dry density of the masses incorporated with granite waste, thereby improving particle packing. However, the dry density of the compositions with granite waste did not increase significantly as the percentage of waste incorporated increased. However, this increase is beneficial in terms of reducing shrinkage and favoring particle consolidation during firing.
- There was an increase in the mechanical strength of the ceramic material with the use of waste. Granite waste increases strength due to its melting action and clay influence.
- The results indicated that the use of ornamental stone waste in the production of red ceramics is feasible and its use should be adjusted for the firing temperature of the pieces. It is an environmentally sound alternative, with the ability to generate a reduction in the order of millions of t of waste that otherwise represents a serious environmental problem.
- Finally, the main objective of this work was to obtain a technical, economic and environmentally viable solution for the waste. The next steps involve the regulation of this waste, with several possibilities for its use in red ceramic. The dissemination, standardization and recommendations for use will transform this waste into a product with equal conditions of use in relation to conventional materials, high added value and the potential for technological innovation.

## Figures and Tables

**Table 1:** Mass formulation.

Compositions of Mass Formulations						
Raw Material	AP	10%	20%	30%	40%	50%
Ceramic Mass	100	90	80	70	60	50
Waste	-	10	20	30	40	50

**Table 2:** Chemical composition of raw materials.

	SiO_2_	Al_2_O_3_	Fe_2_O_3_	Na_2_O	MgO	K_2_O	P_2_O_5_	CaO	Ti_2_O	SO_3_	BaO	LOI *
Ceramic Mass	41.60	30.80	9.00	0.13	1.20	0.92	0.17	0.16	1.30	-	-	14.60
Waste	56.00	19.90	5.80	5.40	1.60	4.30	0.48	3.60	1.10	0.19	0.59	0.74

* LOI, loss on ignition.

**Table 3:** Atterberg limits of ceramic masses.

Ceramic Masses						
Plasticity	PC	10% GW	20% GW	30% GW	40% GW	50% GW
LP	27.7	29.3	29.4	27.8	25.3	23.7
LL	60.1	54.5	51.8	49.2	44.5	43.0
IP	32.4	25.2	22.4	21.3	19.2	19.3

**Table 4:** Analytical results of the leach extract.

	Analysis	Results		Maximum Limit NBR 10004	
Leached	Total Arsenic	<0.0010	mg/L	1	mg/L
	Total Barium	1.107	mg/L	70	mg/L
	Total Cadmium	<0.0010	mg/L	0.5	mg/L
	Total Lead	<0.010	mg/L	1	mg/L
	Total Chrome	<0.010	mg/L	5	mg/L
	Total Mercury	<0.00010	mg/L	0.1	mg/L
	Total Silver	<0.0010	mg/L	5	mg/L
	Total Selenium	<0.010	mg/L	1	mg/L
	Total Fluoride	0.9	mg/L	150	mg/L
	1,1,2-Trichloroethene	<2.00	µg/L	-	µg/L
	1,1-Dichloroethene	<2.00	µg/L	3	mg/L
	1,2-Dichloroethane	<2.00	µg/L	1	mg/L
	1,4-Dichlorobenzene	<2.00	µg/L	7.5	mg/L
	2,4,5-Trichlorophenol	<0.10	µg/L	400	mg/L
	2,4,6-Trichlorophenol	<0.10	µg/L	20	mg/L
	2,4-Dinitrotoluene	<0.010	µg/L	0.13	mg/L
	Benzene	<2.00	µg/L	0.5	mg/L
	Benzo(a)pyrene	<0.010	µg/L	0.07	mg/L
	Vinyl chloride	<2.00	µg/L	0.5	mg/L
	Chlorobenzene	<2.00	µg/L	100	mg/L
	Chloroform	7.89	µg/L	6	mg/L
	Total cresol	<0.010	µg/L	200	mg/L
	Hexachlorobenzene	<0.010	µg/L	0.1	mg/L
	Hexachlorobutadiene	<2.00	µg/L	0.5	mg/L
	Hexachloroethane	<0.010	µg/L	3	µg/L
	Nitrobenzene	<0.010	µg/L	2	mg/L
	Final pH of Leachate	5.25			
	Pyridine	0	µg/L	5	mg/L
	Carbon tetrachloride	<2.00	µg/L	0.2	mg/L
	Tetrachloroethene	<2.00	µg/L	4	mg/L
	2-Methylphenol (o-cresol)	<0.10	µg/L	200	mg/L
	3-Methylphenol, 4-methylphenol (m,p-cresol)	<0.10	µg/L	200	mg/L
	Methyl ethyl ketone	<1.00	µg/L	200	mg/L

**Table 5:** Analytical results of the solubilization extract.

	Analysis	Results		Maximum Limit NBR 10004	
Solubilized	Total Aluminum	0.165	mg/L	0.2	mg/L
	Total Arsenic	0.0024	mg/L	0.01	mg/L
	Total Barium	0.076	mg/L	0.7	mg/L
	Total Cadmium	<0.0010	mg/L	0.005	mg/L
	Total Lead	<0.010	mg/L	0.01	mg/L
	Total Chloride	9	mg/L	250	mg/L
	Total Copper	0	mg/L	2	mg/L
	Total Chrome	<0.010	mg/L	0.05	mg/L
	Total Iron	0	mg/L	0.3	mg/L
	Total Manganese	0.015	mg/L	0.1	mg/L
	Total Mercury	<0.00010	mg/L	0.001	mg/L
	Nitrate (as N)	0.07	mg/L	10	mg/L
	Total Silver	<0.0010	mg/L	0.05	mg/L
	Total Selenium	<0.010	mg/L	0.01	mg/L
	Total Sodium	12.41	mg/L	200	mg/L
	Total Zinc	<0.010	mg/L	5	mg/L
	Total Cyanide	<0.002	mg/L	0.07	mg/L
	Total Phenols	<0.003	mg/L	0.01	mg/L
	Total Fluoride	<0.4	mg/L	1.5	mg/L
	Hexachlorobenzene	<0.010	µg/L	0.001	mg/L
	Final pH of Solubilized	7.42			
	Total Sulfate	46.1	mg/L	250	mg/L
	Surfactants	<0.10	mg/L	0.5	mg/L

## Data Availability

Not applicable.
